# Physical disability, cognition, and depression as determinants of quality of life in multiple sclerosis: a cross-sectional study

**DOI:** 10.3389/fneur.2026.1776488

**Published:** 2026-04-30

**Authors:** Eman M. Khedr, Ahmed A. Karim, Mohammed Y. Ezzeldin, Ahmed Abdelwarith, Gellan K. Ahmed, Nourelhoda A. Haridy

**Affiliations:** 1 Department of Neurology and Psychiatry, Faculty of Medicine, Assiut University, Assiut, Egypt; 2 Department of Neurology and Psychiatry, Faculty of Medicine, Aswan University, Aswan, Egypt; 3 Department of Psychiatry and Psychotherapy, University of Tübingen, Tübingen, Germany; 4 Department of Neurology and Psychiatry, Faculty of Medicine, South Valley University, Qena, Egypt

**Keywords:** cognitive dysfunction, depression, MSIS-29, multiple sclerosis, quality of life

## Abstract

**Background:**

Quality of life (QoL) in multiple sclerosis (MS) is multifactorial, reflecting the combined effects of physical disability, cognitive impairment, and depressive symptoms. While physical disability is a key determinant, the independent contributions of cognition and depression to QoL remain incompletely defined. This study aimed to assess the frequency of cognitive impairment and depressive symptoms in MS and examine their independent impact on QoL beyond physical disability.

**Methods:**

This cross-sectional study included 242 patients with clinically definite MS who were evaluated using standardized clinical and neuropsychological measures. Physical disability was assessed by the Expanded Disability Status Scale (EDSS), motor function by the Nine-Hole Peg Test (9-HPT) and 25-Foot Walk Test (25-FWT), cognition by the Symbol Digit Modalities Test (SDMT), California Verbal Learning Test (CVLT), and Brief Visuospatial Memory Test-Revised (BVMT-R), depression by the Hamilton Depression Rating Scale (HAM-D), and QoL by the MS Impact Scale (MSIS-29). Hierarchical multiple regression was performed to identify independent predictors of total QoL. In this study, cognitive impairment was defined as performance ≥1.5 standard deviations below normative means on at least one cognitive test, and depressive symptoms as HAM-D ≥ 8.

**Results:**

Cognitive impairment was present in 47.9% and depressive symptoms in 61.3% of the cohort. EDSS showed the strongest correlation with MSIS-29 total score (*r* = 0.698, *p* < 0.001), followed by HAM-D (*r* = 0.438, *p* < 0.001) and BVMT-R (*r* = −0.340, *p* < 0.001). In regression analysis, physical disability (*β* = 0.65, *p* < 0.001), depression (*β* = 0.23, *p* < 0.001), and processing speed (*β* = −0.12, *p* = 0.009) were independent predictors of QoL, explaining 64% of its variance.

**Conclusion:**

Although physical disability remains the dominant determinant of QoL in MS, depressive symptoms and cognitive slowing contribute independent and clinically meaningful effects. Routine screening and early intervention for mood and cognitive disturbances are essential to improve overall well-being in patients with MS.

## 1 Introduction

Cognitive dysfunction affects a substantial proportion of people with multiple sclerosis (MS) and is increasingly recognized as a major contributor to reduced everyday functioning and well-being. Beyond motor and sensory deficits, MS affects brain networks involved in cognition and emotional regulation, leading to a range of neuropsychological disturbances that significantly influence patients’ daily lives (1). Accordingly, cognitive impairment and depression are now considered core clinical features of MS rather than merely secondary complications, and they may appear early in the disease course with important implications for long-term outcomes (2–4).

The reported prevalence of cognitive impairment in MS varies widely, ranging from approximately 10% to over 60%, depending on the assessment methods, disease stage, and patient characteristics (5, 6). Several factors have been associated with an increased risk of cognitive decline, including older age, longer disease duration, greater physical disability, and neurodegenerative processes such as brain atrophy and axonal injury (7). Depression is also highly prevalent in MS; a recent meta-analysis estimated a pooled prevalence of 25.3%, with a substantial proportion of patients remaining untreated or experiencing persistent symptoms despite therapy (8).

Both cognitive impairment and depression have been shown to influence health-related quality of life (QoL) significantly in people with MS. Previous studies have demonstrated that depressive symptoms may represent one of the strongest predictors of reduced QoL, sometimes exceeding the impact of physical disability (9). Similarly, cognitive impairment has been consistently associated with poorer QoL outcomes across MS populations (10, 11). These findings suggest that neuropsychological and mood disturbances may substantially shape patients’ perceived well-being and daily functioning.

Despite increasing recognition of these associations, the relative and independent contributions of physical disability, cognitive dysfunction, and depressive symptoms to quality of life (QoL) remain insufficiently characterized in well-defined clinical cohorts. Emerging evidence further suggests that depressive symptoms may partially mediate the relationship between cognitive function and QoL, underscoring the importance of evaluating these domains simultaneously (12, 13). This is particularly relevant in the early stages of the disease, when cognitive decline and mood disturbances may be more responsive to intervention (1).

We hypothesized that cognitive impairment and depressive symptoms would each exert significant and independent negative effects on QoL, even after controlling for physical disability severity and demographic factors. Accordingly, this study aimed to evaluate the relationships among physical disability, cognitive performance, depressive symptoms, and QoL in a large MS cohort using standardized clinical, neuropsychological, and mood assessments. Specifically, we sought to: (1) determine the frequency of cognitive impairment and depression; (2) assess their associations with clinical and demographic variables; and (3) evaluate the independent contributions of physical disability (EDSS), depression (HAM-D), and cognitive performance (SDMT and memory indices) to QoL using hierarchical regression modeling.

## 2 Methods

### 2.1 Study design and participants

This cross-sectional study included 242 consecutive patients with clinically definite MS attending El-Eman Hospital (Assiut, Egypt) and Assiut University Hospital between January 2023 and May 2025.

The inclusion criteria were age ≥18 years, a diagnosis of MS according to the (14) revised McDonald criteria (15), and the ability to complete cognitive and mood assessments. The cohort comprised patients with clinically isolated syndrome (CIS), relapsing–remitting MS (RRMS), secondary progressive MS (SPMS), and primary progressive MS (PPMS) (15).

The exclusion criteria included relapse or corticosteroid treatment within the previous 30 days, major medical or psychiatric comorbidity precluding participation, or insufficient language/cognitive capacity to complete the assessments.

### 2.2 Clinical and demographic variables

All patients received a comprehensive assessment, including demographic and clinical MS characteristics. We recorded age, sex, years of education, disease duration (years), MS phenotype (relapsing vs. progressive), and physical disability, as measured by the Expanded Disability Status Scale (EDSS).

#### 2.2.1 Expanded disability status scale

The EDSS measures neurological disability in MS across eight functional systems, with 0.5-point increments; lower scores indicate mild impairment, and scores >6 reflect severe disability (16). Despite its limitations, the EDSS remains the global standard for MS outcome assessment and cross-trial comparison (17).

#### 2.2.2 9-hole peg test

Hand dexterity was evaluated by timing the placement and removal of nine pegs (18). The standard procedure consists of four trials, two for each hand, using validated test apparatuses (14).

#### 2.2.3 25-foot walk test

Gait speed was assessed by measuring the time required to walk 25 feet at the fastest safe pace (19), with assistive devices permitted as needed (20).

#### 2.2.4 Cognitive assessment

Cognitive function was assessed using the Arabic version of the Brief Cognitive Assessment for Multiple Sclerosis (BICAMS) battery (21), which includes the SDMT (processing speed), CVLT-II (verbal memory), and BVMT-R (visual memory). The total score was used in all analyses. BICAMS was selected for its brief administration (~5 min), high sensitivity to processing-speed deficits typical of MS, and endorsement by expert consensus and the National MS Society as a minimal routine screening tool (22).

Testing was performed in quiet MS outpatient clinics on the morning before MRI acquisition by three trained neurologists (one per center), who had undergone standardized BICAMS training to reduce interrater variability. Scoring was conducted in accordance with the international BICAMS guidelines (22). An age-, sex-, and education-matched healthy control group was used solely to determine normative cut-off values for the cognitive tests. Cut-offs were set at 1.5 SD below the control mean, as recommended previously (21), corresponding to SDMT ≤ 22, CVLT-II ≤ 38, and BVMT-R ≤ 10 (23). Patients were classified as having CI if they scored below these thresholds on at least one test, consistent with commonly applied criteria in MS cognitive research (21, 22, 24, 25).

#### 2.2.5 Depression assessment

Depressive symptoms were evaluated using the 17-item Hamilton Depression Rating Scale (HAM-D), a clinician-rated instrument widely used to assess depression severity. HAM-D scores were interpreted according to standard thresholds: 0–7 indicating no depression, 8–16 mild depression, 17–23 moderate depression, and ≥24 severe depression (26). In the statistical analyses, depression was primarily operationalized as a continuous variable using the total HAM-D score. Additionally, for descriptive purposes, scores ≥8 were considered indicative of clinically significant depressive symptoms. A structured diagnostic interview was not performed.

#### 2.2.6 Quality of life assessment

Disease-specific QoL was assessed using the Multiple Sclerosis Impact Scale (MSIS-29), a 29-item self-administered questionnaire providing physical and psychological subscale scores, with well-established psychometric validity in MS populations (27). The MSIS-29 total score was used as the dependent variable in all analyses, rather than the physical or psychological subscale scores.

#### 2.2.7 Neuroimaging

MS patients underwent MRI on a 1.5 T Philips Achieva scanner. Among the sequences were FLAIR (TR/TE/TI = 9000/120/2500 ms, slice thickness = 3 mm, FOV = 240 mm), T1-weighted (TR/TE = 500/15 ms, slice thickness = 3 mm, FOV = 240 mm), and T2-weighted (TR/TE = 3000/85 ms, slice thickness = 3 mm, FOV = 240 mm). Blinded to the clinical data, two independent radiologists visually identified and evaluated MS lesions. MRI variables were collected to support the diagnosis and clinical characterization of MS. However, they were not included in correlation or regression analyses due to heterogeneity in imaging protocols across centers and the lack of standardized volumetric measurements, which limited comparability across participants.

### 2.3 Ethical approval

The study was approved by the ethical committee of the Faculty of Medicine at Assiut University with Institutional Review Board number (300802). All participants provided written informed consent.

### 2.4 Statistical analysis

All analyses were conducted using IBM SPSS Statistics (version 26). Normality was assessed visually and with the Shapiro–Wilk test. Continuous variables were summarized as mean ± SD or median (interquartile range, IQR) for non-normally distributed data, and categorical variables as counts and percentages. Bivariate associations among BICAMS, HAM-D, MSIS-29 (total and subscales), age, and EDSS were examined using Pearson or Spearman correlation coefficients, as appropriate. The primary outcome was the MSIS-29 total score. Subscale scores were explored descriptively but were not included in regression analyses. Five participants were excluded from regression analyses due to incomplete data on one or more predictor variables, resulting in a final regression sample of *N* = 237.

Hierarchical multiple linear regression was performed to identify determinants of self-reported MS impact (MSIS-29 total score). Variables were entered in theory-driven blocks: Block 1—covariates (gender, age, marital status, working status, education); Block 2 added neurological disability (EDSS); Block 3 added depressive symptoms (HAM-D); Block 4 added processing speed (SDMT). SDMT was selected *a priori* as the representative cognitive variable because it is the most widely used brief screening measure in MS and captures processing speed, a core cognitive domain frequently affected in the disease. Five participants were excluded from regression analyses due to incomplete data on one or more predictor variables, resulting in a final regression sample of *N* = 237. The forced-entry (ENTER) method was applied within each block, with both unstandardized (B) and standardized (*β*) regression coefficients reported. All tests were two-tailed, and statistical significance was set at *p* < 0.05.

## 3 Results

### 3.1 Baseline clinical and radiological characteristics

Table 1 shows the demographic, clinical, radiological, cognitive, and psychiatric characteristics of the MS cohort (*N* = 242). The mean age of participants was 32.1 ± 9.2 years, with a predominance of females (73.3%). The mean disease duration was 4.55 ± 4.14 years, with a diagnostic delay of 2.04 ± 3.23 years. Motor symptoms were the most common onset presentation (49.4%), followed by optic neuritis (23.2%) and sensory symptoms (19.5%). The majority of patients were diagnosed with RRMS (71.5%), while CIS, SPMS, and PPMS accounted for 14.5, 8.3, and 5.8%, respectively. Radiologically, periventricular lesions were the most frequent (95.3%), followed by juxtacortical (66.2%), spinal cord (51.9%), and infratentorial lesions (39.6%).

**Table 1:** Demographic, clinical, and radiological characteristics of the MS cohort (*N* = 242).

Domain	Variable	N	Mean ± SD / n (%)	Range
Demographics	Age (years)	242	32.1 ± 9.2	16–53
	Gender: Male	66 (27.27%)	–	–
	Gender: Female	176 (72.72%)	–	–
	Educational years	242	11.2 ± 4.1	0–19
Clinical	Disease duration (years)	242	4.55 ± 4.14	0.08–20
	Diagnostic delay (years)	242	2.04 ± 3.23	0–15
	Number of attacks	242	3.06 ± 2.17	0–12
	EDSS	242	2.04 ± 1.41	0–9
Symptoms at onset	Sensory	47 (19.5%)	–	–
	Optic neuritis	56 (23.2%)	–	–
	Motor	119 (49.4%)	–	–
	Vertigo	14 (5.8%)	–	–
	Trigeminal neuralgia	3 (1.2%)	–	–
	Headache	2 (0.8%)	–	–
MS subtype	CIS	35 (14.5%)	–	–
	RRMS	173 (71.5%)	–	–
	PPMS	14 (5.8%)	–	–
	SPMS	20 (8.3%)	–	–
MRI lesion location*				
Periventricular lesion	Negative	10 (4.7%)	–	–
	Positive	202 (95.3%)	–	–
Juxtacortical lesion	Negative	70 (33.8%)	–	–
	Positive	137 (66.2%)	–	–
Infratentorial lesion	Negative	125 (60.4%)	–	–
	Positive	82 (39.6%)	–	–
Spinal cord lesion	Negative	99 (46.7%)	–	–
	Positive	110 (51.9%)	–	–
	NA	3 (1.4%)	–	–

*MRI variables are presented for descriptive purposes only and were not included in inferential analyses. CIS, Clinically Isolated Syndrome; RRMS, Relapsing–Remitting Multiple Sclerosis; PPMS, Primary Progressive Multiple Sclerosis; SPMS, Secondary Progressive Multiple Sclerosis.

### 3.2 Physical disability, cognition, quality of life, and depression

Table 2 presents the functional, cognitive, QoL, and depression characteristics of the MS cohort. The mean EDSS was 2.04 ± 1.41, indicating mild disability. The mean 9-HPT and 25-FWT were 29.2 ± 10.9 s and 20.5 ± 13.7 s, respectively, reflecting variable motor performance. Cognitive testing revealed mean SDMT scores of 29.1 ± 10.9, CVLT scores of 39.6 ± 9.2, and BVMT-R scores of 20.3 ± 9.4, indicating cognitive impairment in several domains. The MSIS-29 revealed substantial impact on QoL, with a mean physical score of 40.4 ± 23.1, a psychological score of 18.4 ± 6.3, and a total score of 58.8 ± 25.4. The mean HAM-D was 10.6 ± 7.3. Using the predefined cut-off (HAM-D ≥ 8), more than half of patients (61.3%) met criteria for clinically significant depressive symptoms, most of whom were classified as mild (43.3%), while 11.7% had moderate and 6.3% had severe depression.

**Table 2:** Functional measures, cognitive, QoL, and depression characteristics of the MS cohort (*N* = 242).

Domain	Variable	N	Mean ± SD / n (%)	Range
Functional measures	EDSS	242	2.04 ± 1.41	0–9
	9-HPT (s)	242	29.2 ± 10.9	13.5–132
	25-FWT (s)	242	20.5 ± 13.7	0–90
Cognition	SDMT	242	29.1 ± 10.9	0–57
	CVLT	242	39.6 ± 9.2	6–63
	BVMT-R	242	20.3 ± 9.4	0–36
Cognitive impairment*				
Not impaired	126	52.1%		
Impaired	116	47.9%		
MSIS29	MSIS-29 Physical	242	40.4 ± 23.1	17–126
	MSIS-29 Psychological	242	18.4 ± 6.3	9–44
	MSIS-29 Total	242	58.8 ± 25.4	29–145
Hamilton depression (HAM-D)	Total score	240	10.6 ± 7.3	1–36
Depression status*	Yes	147 (61.3%)		
	No	93 (38.8%)		
Degree of depression	No depression (0–7)	93 (38.8%)		
	Mild (8–16)	104 (43.3%)		
	Moderate (17–23)	28 (11.7%)		
	Severe (≥24)	15 (6.3%)		

*Cognitive impairment is defined as ≥1.5 SD below normative means on at least one cognitive test; depressive severity is categorized according to the HAM-D scores: 0–7 = no depression; 8–16 = mild; 17–23 = moderate; ≥24 = severe. EDSS, Expanded Disability Status Scale; 9-HPT, Nine-Hole Peg Test; 25-FWT, 25-Foot Walk Test; SDMT, Symbol Digit Modalities Test; CVLT, California Verbal Learning Test; BVMT-R, Brief Visuospatial Memory Test–Revised; HAM-D, Hamilton Depression Rating Scale; MSIS-29, Multiple Sclerosis Impact Scale–29.

### 3.3 Clinical and cognitive correlates of quality of life

**Figure 1:**
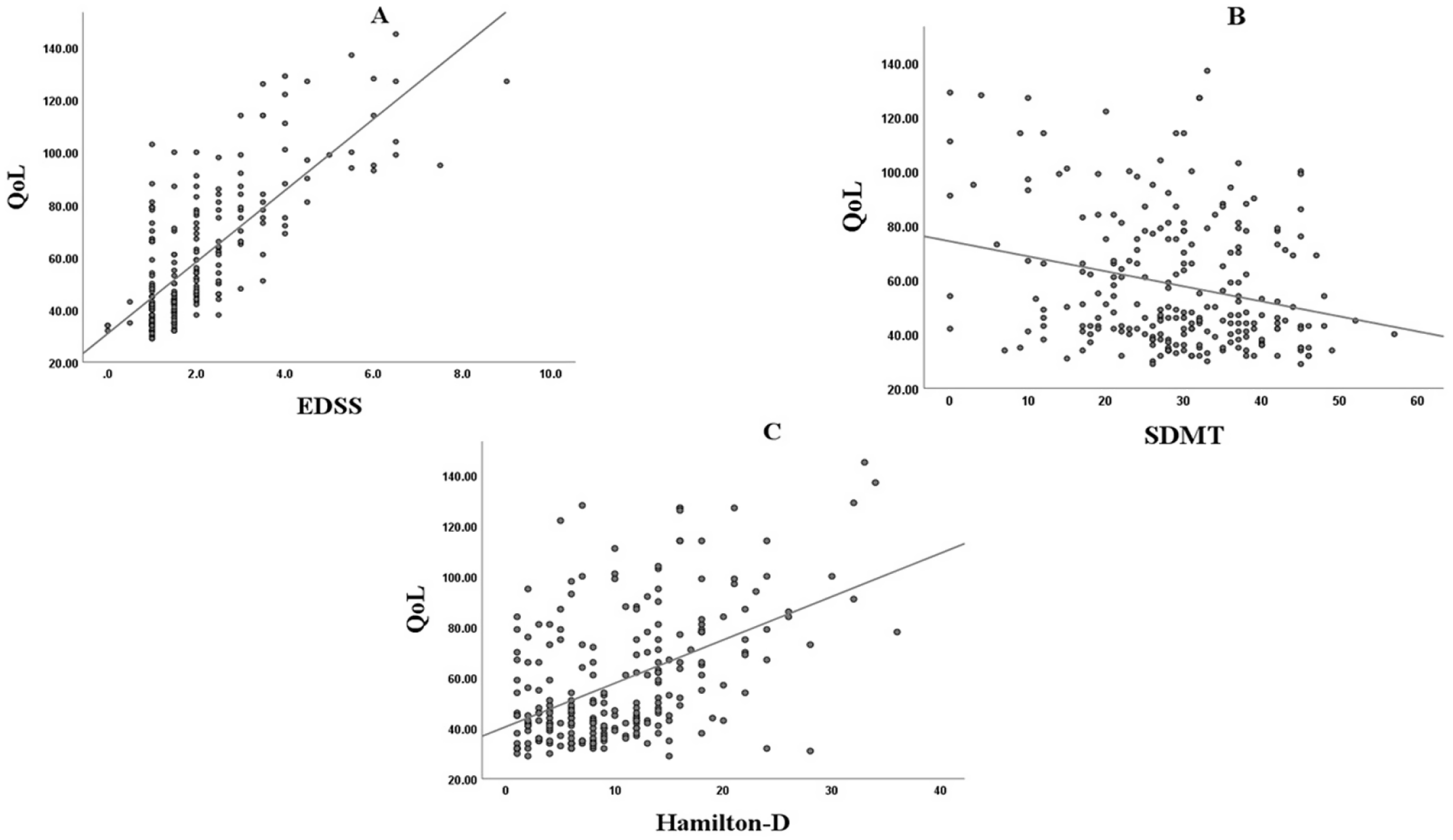
Scatter plots of the principal correlates of MSIS-29 total score in multiple sclerosis. Scatter plots with fitted linear regression lines showing the unadjusted associations between MSIS-29 total score and **(A)** Expanded Disability Status Scale (EDSS), **(B)** Hamilton Depression Rating Scale (HAM-D), and **(C)** Symbol Digit Modalities Test (SDMT). Higher MSIS-29 scores indicate worse quality of life. Correlation coefficients shown in the panels are Spearman’s rho values corresponding to Table 3.

Table 3 presents the correlations between demographic, clinical, cognitive, functional, and psychiatric variables with MSIS-29 quality of life domains. Several strong associations were observed. Physical disability and functional impairment showed the strongest correlations with poorer QoL. Depression severity was strongly linked to psychological QoL, and moderately to physical and total domains. Cognitive performance, particularly BVMT-R and CVLT, demonstrated negative correlations, suggesting that poorer cognition was associated with reduced QoL. Disease burden indicators, such as longer duration, diagnostic delay, and a higher number of attacks, also correlated with impaired QoL (all *p* < 0.001). Demographic variables had weaker but significant effects. Higher education exerted a modest protective effect. Interestingly, motor onset symptoms predicted worse QoL (*r* = 0.236, *p* < 0.01), whereas optic neuritis at onset was modestly protective (*r* = −0.171, *p* < 0.05). These relationships are visually summarized in Figure 1, which shows the strongest positive unadjusted association for EDSS (r_s = 0.698, *p* < 0.001), a moderate positive association for HAM-D (r_s = 0.438, *p* < 0.001), and a weaker inverse association for SDMT (r_s = −0.191, *p* = 0.003).

**Table 3:** Correlations between demographic, clinical, cognitive, functional, and psychiatric variables with MSIS-29 physical, psychological, and total scores.

Variable	Physical	Psychological	Total MSIS-29
Age	*r* = 0.240** ***p* < 0.0001**	*r* = 0.098 *p* = 0.132	*r* = 0.247** ***p* < 0.0001**
Gender	*r* = −0.070 *p* = 0.283	*r* = 0.032 *p* = 0.623	*r* = −0.060 *p* = 0.357
Educational years	*r* = −0.124 *p* = 0.055	*r* = −0.044 *p* = 0.497	*r* = −0.138* ***p* = 0.033**
Disease duration	*r* = 0.383** ***p* < 0.0001**	*r* = 0.175** ***p* = 0.007**	*r* = 0.374** ***p* < 0.0001**
Diagnostic delay	*r* = 0.352** ***p* < 0.0001**	*r* = 0.224** ***p* < 0.0001**	*r* = 0.359** ***p* < 0.0001**
Motor onset	*r* = 0.236** ***p* = 0.006**	*r* = 0.097 *p* = 0.267	*r* = 0.206* ***p* = 0.017**
Optic onset	*r* = −0.171* ***p* = 0.049**	*r* = −0.040 *p* = 0.647	*r* = −0.142 *p* = 0.102
Sensory onset	*r* = 0.097 *p* = 0.265	*r* = 0.045 *p* = 0.607	*r* = 0.086 *p* = 0.323
Cerebellar onset	*r* = 0.045 *p* = 0.607	*r* = 0.116 *p* = 0.183	*r* = 0.079 *p* = 0.366
Number of attacks	*r* = 0.497** ***p* < 0.0001**	*r* = 0.306** ***p* < 0.0001**	*r* = 0.511** ***p* < 0.0001**
EDSS	*r* = 0.664** ***p* < 0.0001**	*r* = 0.444** ***p* < 0.0001**	*r* = 0.698** ***p* < 0.0001**
9-HPT	*r* = 0.491** ***p* < 0.0001**	*r* = 0.301** ***p* < 0.0001**	*r* = 0.492** ***p* < 0.0001**
25-FWT	*r* = 0.615** ***p* < 0.0001**	*r* = 0.314** ***p* < 0.0001**	*r* = 0.612** ***p* = 0.0001**
SDMT	*r* = −0.163* ***p* = 0.012**	*r* = −0.111 *p* = 0.088	*r* = −0.191** ***p* = 0.003**
CVLT	*r* = −0.231** ***p* < 0.0001**	*r* = −0.108 *p* = 0.097	*r* = −0.224** ***p* < 0.0001**
BVMT-R	*r* = −0.324** ***p* < 0.0001**	*r* = −0.169** ***p* = 0.009**	*r* = −0.340** ***p* < 0.0001**
HAM-D	*r* = 0.345** ***p* < 0.0001**	*r* = 0.509** ***p* < 0.0001**	*r* = 0.438** ***p* < 0.0001**

r = Spearman’s rho correlation coefficient. *p < 0.05; **p < 0.01 (2-tailed). EDSS, Expanded Disability Status Scale; 9-HPT, Nine-Hole Peg Test; 25-FWT, 25-Foot Walk Test; SDMT, Symbol Digit Modalities Test; CVLT, California Verbal Learning Test; BVMT-R, Brief Visuospatial Memory Test–Revised; HAM-D, Hamilton Depression Rating Scale; MSIS-29, Multiple Sclerosis Impact Scale–29. Bold values are statistically significant *p*-values.

### 3.4 Predictors of quality of life (hierarchical regression analysis)

Tables 4, 5: A theory-driven hierarchical regression examined predictors of MSIS-29 total scores. Covariates explained 11.0% of the variance (R^2^ = 0.110). Adding EDSS resulted in a substantial increase (R^2^ = 0.583; ΔR^2^ = 0.473), indicating that neurological disability is the dominant contributor. Adding depressive symptoms (HAM-D) yielded a further significant increment (R^2^ = 0.631; ΔR^2^ = 0.048). Finally, adding processing speed (SDMT) provided a small but reliable improvement (R^2^ = 0.642; ΔR^2^ = 0.011). In bivariate analyses, poorer BVMT-R and CVLT performance was associated with worse MSIS-29 scores. However, these were unadjusted findings. In the primary hierarchical regression model, SDMT was entered as the prespecified cognitive marker to assess whether cognition contributed additional variance beyond demographic covariates, EDSS, and depressive symptoms.

**Table 4:** Hierarchical multiple regression predicting MSIS-29 total (*N* = 237*).

Predictor	B	SE B	*β*	t	*p*	95% CI for B
Intercept	23.332	8.094	—	2.883	0.004	7.383, 39.282
Covariates						
Gender	0.420	2.723	0.007	0.154	0.878	−4.946, 5.786
Age (years)	0.124	0.134	0.045	0.927	0.355	−0.140, 0.388
Marital status	2.050	2.452	0.040	0.836	0.404	−2.781, 6.881
Working status	−1.329	2.651	−0.023	−0.501	0.617	−6.552, 3.895
Educational years	0.515	0.280	0.084	1.841	0.067	−0.036, 1.066
Clinical and cognitive						
EDSS	**11.661**	**0.816**	**0.648**	**14.296**	**< 0.001**	**10.054, 13.268**
HAM-D (depression)	**0.807**	**0.150**	**0.232**	**5.388**	**< 0.001**	**0.512, 1.102**
SDMT (processing speed)	**−0.275**	**0.105**	**−0.118**	**−2.621**	**0.009**	**−0.482, −0.068**

*Five participants were excluded from regression analyses due to incomplete data on one or more predictor variables, resulting in a final regression sample of *N* = 237. Block 1 included covariates (gender, age, marital status, working status, and education), which were entered a priori. B, Unstandardized coefficient; SE B, Standard error of B; β, Standardized coefficient (Beta); t, t-value; p, Significance level; 95% CI for B, 95% Confidence Interval for the unstandardized coefficient. EDSS, Expanded Disability Status Scale; HAM-D, Hamilton Depression Rating Scale; SDMT, Symbol Digit Modalities Test. Bold values are statistically significant *p*-values.

**Table 5:** Model summary and block-wise change.

Block (entered)	R^2^	ΔR^2^	Adj. R^2^	SEE	F-change (df1, df2)	*p*
1. Covariates	0.110	—	0.091	24.275	5.710 (5, 231)	**< 0.001**
2. + EDSS	0.583	0.473	0.572	16.645	261.304 (1, 230)	**< 0.001**
3. + HAM-D	0.631	0.048	0.620	15.689	29.871 (1, 229)	**< 0.001**
4. + SDMT	0.642	0.011	0.630	15.492	6.871 (1, 228)	**0.009**

Binary/dummy variables are coded as in the analytic dataset (0/1). Block 1 included covariates (gender, age, marital status, working status, and education), which were entered a priori. EDSS, Expanded Disability Status Scale; HAM-D, Hamilton Depression; SDMT, Symbol Digit Modalities Test. SEE, standard error of estimate. Durbin–Watson = 1.861. Multicollinearity was acceptable (all VIF ≤ 1.51; all tolerance ≥ 0.66). Bold values are statistical significant *p*-values.

In the final model, EDSS (B = 11.66, *β* = 0.65, *p* < 0.001, 95% CI [10.05, 13.27]) and HAM-D (B = 0.81, *β* = 0.23, *p* < 0.001, 95% CI [0.51, 1.10]) were independent predictors; SDMT showed a modest negative association (B = −0.28, *β* = −0.12, *p* = 0.009, 95% CI [−0.48, −0.07]). Covariates (gender, age, marital and working status, and educational years) were not significant after clinical variables were entered. Model diagnostics were satisfactory, with a Durbin–Watson statistic of 1.861, and multicollinearity indices well within recommended thresholds (all VIF ≤ 1.51). Sensitivity checks identified a small number of higher-leverage observations (defined by predefined thresholds), but excluding these cases did not materially alter the magnitude or significance of the EDSS, HAM-D, or SDMT effects in supplementary analyses.

## 4 Discussion

In this cohort of 242 individuals with MS, we examined the relative contributions of neurological disability, depressive symptoms, and cognitive function to QoL. Our findings demonstrate that QoL in MS is shaped by multiple interacting domains. Physical disability emerged as the strongest determinant of QoL; however, depressive symptoms and cognitive processing speed also contributed independently to patient-reported outcomes. Hierarchical regression analysis showed that EDSS explained the largest proportion of variance in MSIS-29 scores (ΔR^2^ = 0.473), while depressive symptoms (HAM-D) and processing speed (SDMT) provided additional explanatory value (ΔR^2^ = 0.048 and ΔR^2^ = 0.011, respectively). In the final adjusted model, EDSS and HAM-D were the strongest predictors, whereas SDMT demonstrated a modest but significant independent association. These findings highlight the multidimensional determinants of QoL in MS and emphasize that mood and cognitive function contribute unique explanatory power beyond physical disability.

### 4.1 Clinical characteristics of the study cohort

An important characteristic of our cohort was the clinical composition of the study population. Motor symptoms were the most common presenting manifestation in our sample. Although sensory and visual presentations are also frequently reported in MS, motor onset is commonly encountered in hospital-based cohorts and may reflect referral patterns or differences in healthcare-seeking behavior. In addition, the proportion of patients with SPMS in our cohort was relatively low. This likely reflects the relatively young age of the cohort, the modest mean disease duration, and the overall mild disability level observed in this sample. Consequently, the study population primarily represents individuals in earlier disease stages, which should be considered when comparing these findings with cohorts enriched for progressive disease.

### 4.2 Burden of cognitive impairment and depressive symptoms

The present study highlights a considerable neuropsychological burden among patients with MS. Nearly half of the participants demonstrated cognitive impairment (47.9%), while depressive symptoms were reported in 61.3% of the cohort. These findings are consistent with previous reports indicating that cognitive impairment affects approximately 40–65% of individuals with MS (1), underscoring the high prevalence of cognitive dysfunction in this population. Depression is likewise one of the most frequent psychiatric comorbidities in MS; approximately 25–50% of patients develop major depression during their lifetime, two to five times higher than rates observed in the general population (3). Reported prevalence rates vary substantially across studies, ranging from 4.27 to 59.6%, which may reflect differences in study populations, diagnostic criteria, and assessment methods (28).

Emerging longitudinal evidence suggests that depressive symptoms may evolve alongside certain cognitive domains in MS, indicating overlapping neuropsychological manifestations of the disease (29). The high frequency of both cognitive impairment and depressive symptoms observed in the present cohort, therefore, reflects a significant dimension of disease burden that extends beyond physical disability.

Collectively, these findings emphasize the importance of systematic screening for cognitive and psychological symptoms in MS. Recent developments in digital screening tools, including adaptations of the digital BICAMS-based Multiple Screener© (30), may facilitate efficient identification of these commonly underrecognized manifestations, which are increasingly recognized as key determinants of patients’ QoL (3, 31).

### 4.3 Physical disability as the primary determinant of QoL

The current study confirms that neurological disability is an important determinant of quality of life in MS. The observed association between EDSS and MSIS-29 scores reflects the impact of physical impairment on mobility, independence, and daily functioning. These findings are consistent with recent evidence showing that higher disability levels, as measured by EDSS, are consistently associated with poorer patient-reported QoL across both physical and psychological domains in patients with MS, even after accounting for other clinical and demographic factors (32).

However, disability alone does not fully explain QoL impairment. In the current study, hierarchical regression analysis showed that depressive symptoms and slowed processing speed, as measured by SDMT, contributed independently to QoL beyond the effects of physical disability. This aligns with previous reports demonstrating that both depression and cognitive dysfunction are important determinants of patient-reported QoL in MS (12, 33, 34).

### 4.4 Cognitive function and its contribution to quality of life

Cognitive function in the current cohort was significantly associated with QoL. Processing speed, measured by the SDMT, contributed independently to QoL after adjusting for disability and depressive symptoms. Although the effect size was smaller than that of EDSS or depressive symptoms, the persistence of the SDMT–QoL association suggests that slowed information processing affects everyday functioning through mechanisms not fully captured by physical disability scales. Deficits in processing speed may impair multitasking, information handling, and social or occupational performance, thereby influencing patient-perceived functioning. These results are consistent with prior studies identifying SDMT as a sensitive cognitive marker linked to real-world outcomes, including employment status, social participation, and patient-reported QoL (33, 34).

Beyond processing speed, memory performance also demonstrated meaningful relationships with QoL. In bivariate analyses, both BVMT-R and CVLT scores correlated moderately with MSIS-29 outcomes, supporting evidence that deficits in memory and visuospatial learning can influence patient-reported functioning in MS. Gómez-Melero et al. highlighted the relevance of multiple cognitive domains in determining functional outcomes (1), while Chruzander et al. showed in a 10-year longitudinal study that cognitive impairment independently predicts poorer QoL over time (10). Although informative, the primary adjusted model was intentionally specified using SDMT as the representative cognitive measure; accordingly, these memory findings should be interpreted as unadjusted associations rather than independent multivariable effects.

### 4.5 Depression and quality of life

Depressive symptoms showed particularly strong associations with psychological QoL in our cohort. HAM-D scores correlated strongly with MSIS-29 scores and remained significant in multivariable analyses, highlighting the pervasive impact of mood disturbances on patient-perceived well-being. These findings are consistent with earlier observations that depression is among the most powerful psychosocial determinants of QoL in MS (13, 35, 36). Similarly, Zhang et al. demonstrated in a large cohort of nearly 2,000 patients that depression was the strongest predictor of health-related QoL across MS phenotypes (9). Longitudinal studies further demonstrate that fluctuations in depressive symptoms closely track changes in QoL over time (37), reinforcing the notion that mood represents a critical therapeutic target in the comprehensive management of MS.

### 4.6 Clinical implications

These findings have important implications for clinical practice. Evidence from systematic reviews indicates that psychological interventions, including cognitive-behavioral therapy and structured self-management programs, can improve QoL in people with MS (11). Early identification and management of modifiable factors, such as depression and cognitive impairment, may reduce their long-term impact on patient well-being (10).

The current study reinforces the importance of routine multidimensional screening using validated instruments for both mood and cognitive assessment. Reliance solely on physical disability scales, such as EDSS, risks overlooking two major modifiable contributors to QoL—depression and cognitive dysfunction. Early detection, timely intervention, and cognitive rehabilitation may therefore enhance patient-perceived well-being even in the absence of measurable physical improvement.

While disease-specific instruments such as the MSIS-29 provide detailed insight into the patient experience, their length may limit routine implementation in busy clinical settings. Recent approaches employing brief generic patient-reported outcome measures may improve feasibility and facilitate systematic QoL monitoring across neurological disorders, supporting more individualized patient care.

### 4.7 Strengths and limitations

The present study has several methodological strengths. The relatively large sample size (*n* = 242) allowed simultaneous evaluation of multiple determinants of QoL using hierarchical regression modeling. The study combined objective cognitive measures (SDMT, CVLT, BVMT-R), validated mood assessment (HAM-D), standardized functional tests (EDSS, 9-HPT, 25-FWT), and clinical disease burden indicators. This comprehensive approach enabled a detailed examination of the independent contributions of disability, cognition, and mood to QoL.

Several limitations should be considered when interpreting these findings. First, the cross-sectional design precludes conclusions about causal relationships among disability, cognition, depression, and quality of life. Longitudinal studies are needed to clarify the temporal interactions between these domains. Second, MRI data were collected primarily to characterize lesion distribution and were not incorporated into the regression analyses. Quantitative volumetric measures, such as whole-brain or regional atrophy (e.g., cortical or thalamic), were not systematically available; their absence limits the mechanistic interpretation, as volumetric metrics are more strongly associated with cognitive and affective dysfunction in MS than lesion counts alone.

Third, the clinical composition of the cohort may have influenced some observed associations. Motor symptoms were the most frequent onset presentation and showed a modest association with poorer QoL. While this may reflect the functional impact of early motor impairment on mobility and independence, it may also be partly related to the distribution of onset phenotypes within the sample. Finally, the cohort was characterized by relatively mild disability (low mean EDSS) and a small proportion of patients with SPMS, likely reflecting the relatively early disease stage of this outpatient population. Although this allowed the evaluation of cognitive and mood factors before the development of advanced disability, it may limit generalizability to cohorts with more advanced or progressive disease. Future longitudinal studies using standardized volumetric MRI and repeated cognitive and mood assessments are needed to clarify causal pathways, identify structural correlates, and validate the observed associations of impaired quality of life in more diverse MS populations.

## 5 Conclusion

QoL in MS is influenced by a complex interplay between physical disability, depressive symptoms, and cognitive impairment. Although neurological disability remains the dominant determinant, depressive symptoms and slowed cognitive processing independently contribute to poorer patient-reported outcomes. Memory measures were associated with reduced QoL in unadjusted analyses. These findings emphasize the importance of a holistic clinical approach that addresses psychological and cognitive domains alongside traditional neurological assessment to optimize QoL for people living with MS.

## Data Availability

The original contributions presented in the study are included in the article/supplementary material, further inquiries can be directed to the corresponding authors.
